# Impact of Oncotype DX testing on ER+ breast cancer treatment and survival in the first decade of use

**DOI:** 10.1186/s13058-021-01453-4

**Published:** 2021-07-17

**Authors:** Evelien Schaafsma, Baoyi Zhang, Merit Schaafsma, Chun-Yip Tong, Lanjing Zhang, Chao Cheng

**Affiliations:** 1 Department of Molecular and Systems Biology, Dartmouth College, Hanover, NH 03755 USA; 2 Department of Chemical and Biomolecular Engineering, Rice University, Houston, TX 77030 USA; 3 Faculty of Medical Sciences, University of Groningen, Groningen, The Netherlands; 4 Department of Medicine, Baylor College of Medicine, Houston, TX 77030 USA; 5 Department of Biological Sciences, Rutgers University Newark, Newark, NJ USA; 6 Department of Pathology, Princeton Medical Center, Plainsboro, NJ USA; 7 Dan L Duncan Comprehensive Cancer Center, Baylor College of Medicine, Houston, TX 77030 USA; 8 Department of Biomedical Data Science, Geisel School of Medicine at Dartmouth, Lebanon, NH 03756 USA; 9 The Institute for Clinical and Translational Research, Baylor College of Medicine, Houston, TX 77030 USA

**Keywords:** Oncotype DX, Breast cancer, Prognosis, Adjuvant chemotherapy

## Abstract

**Background:**

The Oncotype DX breast recurrence score has been introduced more than a decade ago to aid physicians in determining the need for systemic adjuvant chemotherapy in patients with early-stage, estrogen receptor (ER)+, lymph node-negative breast cancer.

**Methods:**

In this study, we utilized data from The Surveillance, Epidemiology, and End Results (SEER) Program to investigate temporal trends in Oncotype DX usage among US breast cancer patients in the first decade after the introduction of the Oncotype DX assay.

**Results:**

We found that the use of Oncotype DX has steadily increased in the first decade of use and that this increase is associated with a decreased usage of chemotherapy. Patients who utilized the Oncotype DX test tended to have improved survival compared to patients who did not use the assay even after adjusting for clinical variables associated with prognosis. In addition, chemotherapy usage in patients with high-risk scores is associated with significantly longer overall and breast cancer-specific survival compared to high-risk patients who did not receive chemotherapy. On the contrary, patients with low-risk scores who were treated with chemotherapy tended to have shorter overall survival compared to low-risk patients who forwent chemotherapy.

**Conclusion:**

We have provided a comprehensive temporal overview of the use of Oncotype DX in breast cancer patients in the first decade after Oncotype DX was introduced. Our results suggest that the use of Oncotype DX is increasing in ER+ breast cancer and that the Oncotype DX test results provide valuable information for patient treatment and prognosis.

**Supplementary Information:**

The online version contains supplementary material available at 10.1186/s13058-021-01453-4.

## Background

Breast cancer is the most common type of cancer in women in the world and the second most common cancer overall [1]. The incidence of breast cancer has been increasing by 3.1% every year and this trend is expected to continue [2]. Approximately 70% of all breast cancer patients presents with ER+ human epidermal growth factor 2 (HER2)− breast cancer [3, 4], which has a 5-year overall survival rate of > 94% [5]. This high survival rate is partially due to the low rate of breast cancer recurrence following treatment of adjuvant chemotherapy in ER+HER2−, node-negative breast cancer after surgical resection [6–8]. However, since only ~ 15% of ER+HER2− breast cancer patients would experience recurrence at 5 years in the absence of adjuvant chemotherapy [8], 85% of patients may be exposed to chemotherapy toxicity with minimal clinical benefit. To decrease the overuse of adjuvant chemotherapy, patient stratification based on breast cancer recurrence risk has become an effective strategy to aid in individualized breast cancer treatment [9, 10].

The Oncotype DX assay has been used widely to predict the recurrence risk of breast cancer after surgical resection in ER+HER2− breast cancer [11]. Based on the expression levels of 21 genes, the test calculates a risk recurrence score from 0 to 100 that stratifies patients into three groups: high- (≥ 31), intermediate- (18–30), and low-risk (0–17) of recurrence. The Oncotype DX test has been endorsed by the American Society of Clinical Oncology (ASCO) and the National Comprehensive Cancer Network (NCCN) [11, 12]. The NCCN recommends the use of Oncotype DX for ER+HER2−, early-stage (T1 or T2), lymph node-negative (pN0) breast cancer. Based on the RxPONDER trial (NCT01272037) [13, 14], patients with ER+HER2−, early-stage, lymph node-positive (pN+) breast cancer also derive benefit from the Oncotype Dx assay. Since the formal validation of the Oncotype DX assay in 2004 [9], the uptake of testing has increased each year [15].

A number of studies have evaluated chemotherapy usage after Oncotype DX test results [16]. Consistently, patients with low-risk Oncotype DX scores do not benefit from the additional chemotherapy and are consequently rarely treated with chemotherapy [16–19]. On the contrary, the usage of chemotherapy is reported highest among patients with an Oncotype DX score ≥ 31 (high risk of recurrence) compared to lower Oncotype DX scores [16, 19–21]. High-risk patients identified by the Oncotype DX assay are more likely to benefit from chemotherapy than low-risk patients in both neoadjuvant and adjuvant treatment regimens [22]. The benefit of chemotherapy in the intermediate-risk group is less clear. A large randomized trial (TAILORx) suggested that disease-free and overall survival are similar between intermediate-risk patients randomly stratified into no adjuvant treatment or adjuvant chemotherapy [23]. Other factors that are considered when deciding if adjuvant chemotherapy is recommended include the size of the tumor, lymph node status, differentiation, time period of treatment, and patient age [24, 25]. For example, approximately 90% of patients aged < 40 with a high recurrence score were treated with chemotherapy, whereas only 50% in the elderly (> 80 years) population was treated with chemotherapy [26].

In this study, we performed a systematic analysis on the Surveillance, Epidemiology, and End Results (SEER) breast cancer dataset [27]. We aimed to provide a comprehensive overview of Oncotype DX testing rates and how these are related to different clinical variables. Although prognostic relationships between Oncotype DX scores and patient survival have been studied, we wanted to provide a year-by-year overview of prognostic associations while also adjusting for important clinical variables to evaluate if prognostic trends could be observed. These analyses were conducted with the ultimate goal of providing useful information to refine the guidelines for directing Oncotype DX tests.

## Materials and methods

### SEER-Oncotype DX database

The SEER-Oncotype DX data for breast cancer patients diagnosed between 2004 and 2015 were used in this analysis. These data were generated by linking Oncotype DX test results with invasive breast cancer cases from 17 SEER registries [27]. The SEER database provides patient clinical information including year of diagnosis (“Year.of.diagnosis”), age (“Age.at.diagnosis”), race (“Race.ethnicity”), tumor type (“Breast.Subtype.2010”), grade (“Grade”), stage (“Breast.Adjusted.AJCC.6^th^.Stage.1988.2015”), and ER status (“ER.Status.Recode.Breast.Cancer.1990”). Breast cancer-specific survival (BCSS) (“SEER.cause.specific.death.classification”) and overall survival (OS) (“Vital.status.recode.study.cutoff.used”) were also provided. For patients who underwent Oncotype DX testing, test results were provided as continuous recurrence-risk scores (“Oncotype.DX.Breast.Recurrence.Score”) and according to risk categories provided by SEER: high (risk score > 30), intermediate (risk score 18–30), and low-risk (risk score < 18) (“Oncotype.DX.RS.group.RS.18.RS.18.30.RS.30”). In this study, we focused solely on ER+ breast cancer patients aged 35 to 80 years at the time of diagnosis. Of note, patients with ER+HER2+ breast cancer were patients whose tumors were HER2+ according to SEER data but the Oncotype DX report indicated HER2− status per RT-PCR. Consequently, the ER+HER2+ patient group described in this study is a subgroup of those with ER+HER2+ breast cancer and does not resemble all patients with ER+HER2+ breast cancer. Patients with precancerous disease (stage = 0) were excluded from the study. The final data contained a total of 375,350 unique patient IDs. Among these patients, 89,255 patients (24%) underwent Oncotype DX testing with 6414 (7%), 31,302 (25%), and 51,539 (58%) high-, intermediate-, and low-risk test results, respectively. All exact SEER variable names have been provided in quotation marks above.

### Survival analysis

Survival analyses were performed using the R survival package (version 3.2-7). Log-rank tests were performed to evaluate overall or breast cancer-specific survival probabilities between two groups, using the “survdiff” function. Univariate Cox proportional hazards regression was performed on continuous Oncotype DX scores using the “coxph” function. Multivariate Cox proportional hazards regression was performed similarly while adjusting for age, tumor stage, lymph node status, and breast cancer subtype for years 2010–2015. Hazard ratios were extracted from both univariate and multivariate models for Oncotype DX scores. Kaplan-Meier plots were generated using the “survfit” function from the R survival package.

### Statistical analyses

All analyses were performed in R (version 3.6.2). Descriptive statistics were used to summarize the SEER data according to patient and tumor characteristics. T-test and chi-squared test was performed to determine the differences in continuous and categorical patient characteristics between Oncotype DX usage (user vs. non-user). All tests were two-sided if applicable and statistical significance was assessed using an alpha of 0.05.

## Results

### Application of Oncotype DX test in ER+ breast cancer patients

Utilizing the SEER database, we first defined clinical differences among 375,350 breast cancer patients who were stratified based on the usage of Oncotype DX (users vs. non-users) (Table 1). Multivariable analysis indicated that patients with lower tumor stages, lower grade, negative lymph node status, negative HER2 status, and of Caucasian descent were all significantly more likely to be an Oncotype DX user compared to non-users (Table 1). Discrepancies in Oncotype DX testing rate between different races have indeed been observed in previous studies [20, 28]. Oncotype DX users were also less likely to have received chemotherapy, but they were more likely to have received radiation therapy compared to non-Oncotype DX users.

**Table 1:** Patient characteristics of the SEER-Oncotype cohort. *P* values were calculated by t-test and chi-squared test for continuous and categorical characteristics, respectively

	Users (***n*** = 89,255)	Non-users (***n*** = 286,095)	***P*** value
Mean age (SD)	57.8 (10.0)	59.5 (11.5)	<1e−314
Stage (%)			<1e−314
I	58,512 (65.6)	132,415 (46.3)	
II	29,494 (33.0)	97,347 (34.0)	
III	1104 (1.2)	40,265 (14.1)	
IV	145 (0.2)	16,068 (5.6)	
Grade (%)			<1e−314
I	70,648 (26.1)	25,215 (28.9)	
II	126,526 (46.8)	47,690 (54.7)	
III	73,256 (27.1)	14,338 (16.4)	
Lymph node (%)			<1e−314
Negative	76,401 (85.6)	174,024 (60.8)	
Positive	12,848 (14.4)	109,956 (38.4)	
Unknown	6 (0.0)	2115 (0.8)	
HER2 (%) ^**a**^			<1e−314
Negative	62,115 (95.3)	112,187 (78.2)	
Positive	1199 (1.8)	24,469 (17.1)	
Unknown	1889 (2.9)	6850 (4.7)	
Race (%)			3.E−71
White	73,722 (82.6)	230,090 (80.4)	
Black	7129 (8.0)	69,307 (10.0)	
Asian	6837 (7.7)	22,592 (7.9)	
Other	1293 (1.4)	3962 (1.4)	
Unknown	274 (0.3)	851 (0.3)	
Chemotherapy (%)			<1e−314
No/unknown	67,853 (77.7)	159,334 (55.7)	
Yes	19,948 (22.3)	126,761 (44.3)	
Radiation therapy (%)			7.E−313
No/unknown	34,763 (38.9)	132,040 (46.2)	
Yes	54,492 (61.1)	154,055 (53.8)	

^a^ Since only samples after 2009 contained breast cancer subtype information, the displayed percentages include patients from 2010 to 2015 only

**Fig. 1:**
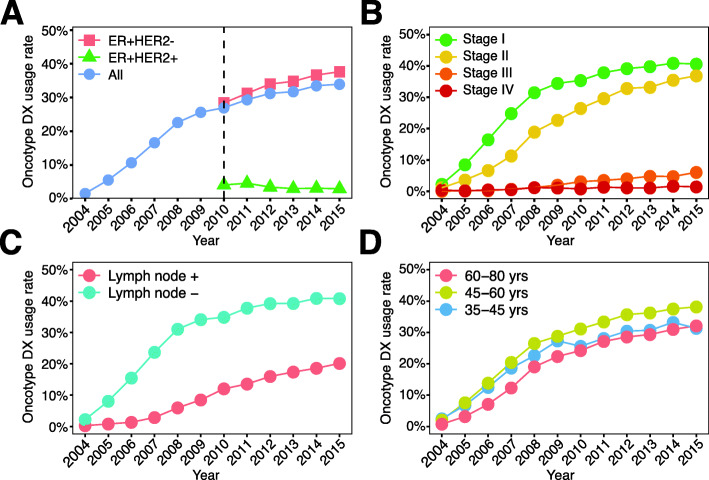
Clinical characteristics of Oncotype DX users between 2004 and 2015. **A** Oncotype DX usage rate among patients with ER+HER2−, ER+HER2+, and all breast cancer subtypes. **B** Oncotype DX usage rate among the four stages of breast cancer. **C** Oncotype DX usage rate between patients with lymph node-positive and negative breast cancer. **D** Oncotype DX usage rate among different patient age groups. Usage rates were calculated based on the total number of patients within each indicated patient subgroup

The usage of Oncotype DX steadily increased over the last decade with 34% of all ER+ breast cancers undergoing testing in 2015 (Fig. 1A). The percentage of patients with ER+HER2+ breast cancer who underwent testing remained relatively stable around 3% of all ER+HER2+ breast cancer patients. Notably, ER+HER2+ patients in this cohort were classified as HER2+ by SEER but showed HER2 negativity according to Oncotype DX HER2 testing. Thus, this patient group likely resembles a subset of ER+HER2+ patients. Consequently, our reported testing rate of 3% is likely an underestimation of the true testing rate in ER+HER2+ breast cancer. Most Oncotype DX users had early-stage disease (Fig. 1B), consistent with current Oncotype DX guidelines [14]. However, a slightly increasing trend in Oncotype DX testing was observed in stage III breast cancer patients, starting from 0% in 2004 to 6% in 2015 (Fig. 1B). Tumors with lower tumor (grade I and II) were more likely to use Oncotype DX testing compared to high grade (grade III) (Additional file 1 – Figure 1A). Oncotype DX usage increased over time in both lymph node-negative and positive breast cancer (Fig. 1C). The usage of Oncotype DX in lymph node-negative breast cancer patients was more than twice the usage of Oncotype DX in lymph node-positive breast cancer (Fig. 1C). Lastly, differences in Oncotype DX usage were observed between different age groups. Older breast cancer patients (60–80 years) were least likely to utilize Oncotype DX throughout the entire study period (Fig. 1D), whereas middle-aged patients (45–60 years) were most likely to utilize Oncotype DX (Fig. 1D). Although young breast cancer patients (35–45 years) utilized Oncotype DX at the same rate as middle-aged patients in the first few years after the introduction of Oncotype DX (2004–2007), their usage in the latter part of the study period was almost identical to older patients with breast cancer (Fig. 1D).

### Oncotype DX testing is associated with decreased usage of chemotherapy

**Fig. 2:**
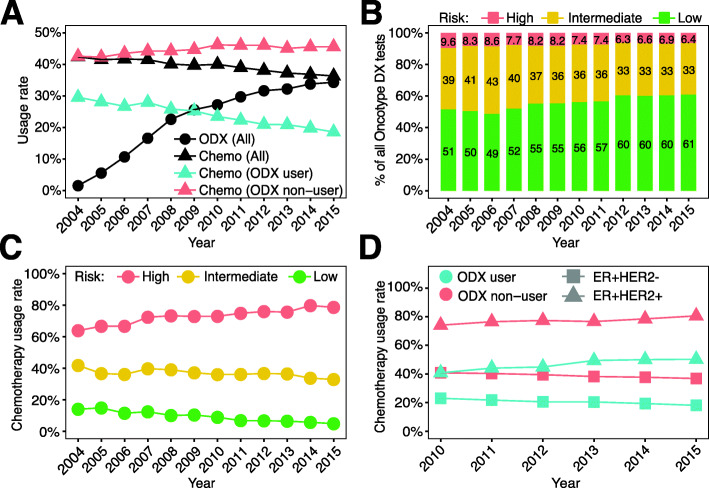
Usage of chemotherapy in Oncotype DX users between 2004 and 2015. **A** Oncotype DX usage rates among patients undergoing chemotherapy (triangles) and overall Oncotype DX usage rate (circles). **B** Percentage of risk groups (high-, intermediate-, and low-risk) classified by Oncotype DX test results. **C** Percentage of chemotherapy usage between high-, intermediate-, and low-risk Oncotype DX groups. **D** Chemotherapy usage among patients with ER+HER2− and ER+HER2+ breast cancer, stratified by Oncotype DX usage. For **A**, **C**, and **D**, usage rates were calculated based on the total number of patients within each indicated patient subgroup

An important application of the Oncotype DX assay is to aid physicians in deciding whether adjuvant chemotherapy would likely benefit a patient with breast cancer. We therefore investigated the relationship between Oncotype DX testing and chemotherapy usage. As shown, from 2004 to 2015, the use of Oncotype DX in ER+ breast cancer patients increased steadily from 1.5% to 34% (Fig. 2A). Meanwhile, the rate of chemotherapy usage continuously decreased from 42 to 36% (Fig. 2A). This trend is likely related to the decrease in chemotherapy usage in patients who underwent Oncotype DX testing; the rate of chemotherapy usage continuously decreased in Oncotype DX users, but continuously increased in non-users from 2004 to 2015 (Fig. 2A).

With more ER+ breast cancer patients utilizing the Oncotype DX assay, an overall decrease in the percentage of high-risk patients (from 9.6% in 2004 to 6.3% in 2015) and an increase in the percentage of low-risk patients (from 51% in 2004 to 61% in 2015) were observed (Fig. 2B). These percentages are similar to previously reported percentages [29–31]. When examining the usage of chemotherapy in the three risk groups, we observed that the chemotherapy rate continuously increased in the high-risk group, but slowly decreased year by year in the intermediate- and low-risk groups (Fig. 2C). This is consistent with the guidelines of Oncotype DX, which recommend adjuvant chemotherapy for ER+ breast cancer patients with high recurrence risk and no adjuvant chemotherapy for patients with low risk of recurrence [14]. Chemotherapy was more frequently used to treat patients with ER+HER2+ compared to ER+HER2− breast cancer (Fig. 2D). In both of these breast cancer subtypes, Oncotype DX users showed a lower rate of chemotherapy usage compared to non-users.

## Oncotype DX users tend to have better prognosis

**Fig. 3:**
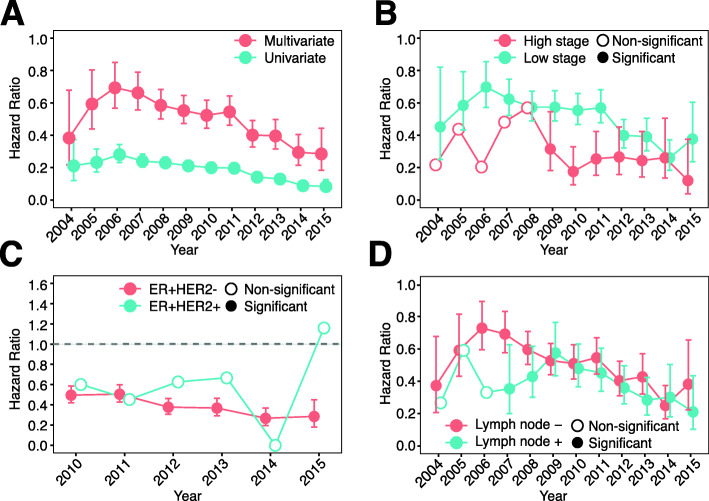
Use of Oncotype DX improves survival. **A** Hazard ratio of Oncotype DX usage between 2004 and 2015 from univariate and multivariable Cox models. Multivariable Cox models were adjusted for tumor stage, tumor grade, lymph node status, breast cancer subtype (ER+HER2− or ER+HER2+), and patients’ age. **B** Hazard ratios of Oncotype DX usage stratified by tumor stage between 2004 and 2015 from univariate Cox regression models. **C** Hazard ratio of Oncotype DX usage stratified by subtypes between 2010 and 2015 from univariate Cox regression models. **D** Hazard ratio of Oncotype DX usage stratified by lymph node status between 2004 and 2015 from univariate Cox regression models. P values below 0.05 were considered significant

To examine the potential benefit from Oncotype DX test results, we compared the survival time between Oncotype DX users and non-users. Univariate Cox regression analysis indicated that users tended to have significantly longer BCSS compared to non-users in all years from 2004 to 2015 (Fig. 3A). Relative to non-users, users showed HRs around 0.2, indicating an 80% lower risk of breast cancer-specific death. According to the guidelines, Oncotype DX tests are recommended for ER+ patients with lower tumor stages and negative lymph node status [14], which are clinical characteristics that are known to be associated with good prognosis. Thus, multivariable Cox regression models were used to adjust for tumor stage and lymph node status, as well as for patients’ age. As shown, after adjustment, Oncotype DX test users still demonstrated significantly longer survival times compared to nonusers (Fig. 3A). The overall benefit from genomic testing continuously increased from 2006 to 2015 with hazard ratios trending down over this time period.

To obtain a more detailed association between Oncotype DX usage, patient prognosis, and clinical variables, we next stratified patients based on tumor stage. A significant benefit from the Oncotype DX test was observed for both late stage (2009–2015) and early-stage (in all years) in ER+ breast cancer patients (Fig. 3B). Without considering other clinical variables, patients with late-stage disease seemed to benefit more from Oncotype DX testing, showing lower hazard ratios compared to early-stage disease (Fig. 3B). The prognosis of patients with low and high grade tumors was identical, showing comparable hazard ratios for almost all evaluated years (Additional file 1 – Figure 1B). For patients with ER+HER2− breast cancer, Oncotype DX users showed consistent benefit with significant hazard ratios in all evaluated years (Fig. 3C). Patients with ER+HER2+ breast cancer seemed to also benefit from Oncotype DX testing, but significantly longer BCSS was not observed compared to non-users in all years (Fig. 3C). In addition, both lymph node-positive (in all years) and negative (2007–2015) patients benefited from the Oncotype DX testing (Fig. 3D). Taking together, our results indicate that patients with ER+HER2− breast cancer demonstrated significant benefit from the Oncotype DX test regardless of tumor stage and lymph node status.

### Oncotype DX score is predictive of patient survival

**Fig. 4:**
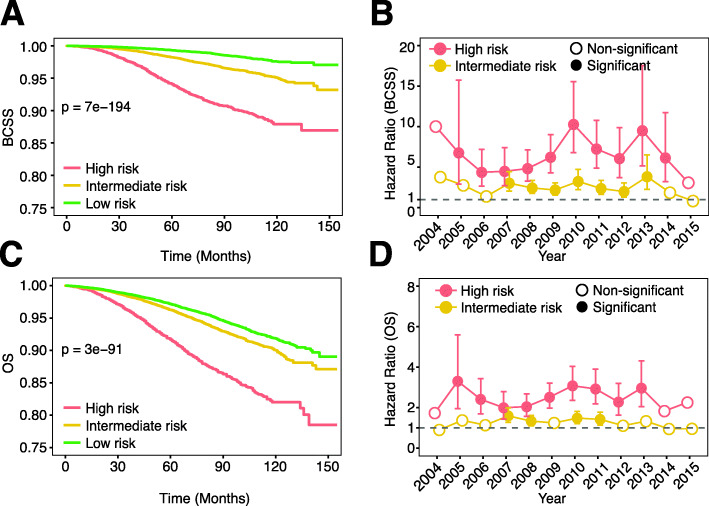
Oncotype DX score is predictive of patient’s survival. **A** Survival plot of 3 risk groups classified by Oncotype DX (BCSS). **B** Hazard ratio of high and intermediate-risk groups using low-risk as baseline (BCSS). Multivariable Cox models were adjusted for tumor stage, lymph node status, breast cancer subtype (ER+HER2− or ER+HER2+), and patients’ age. **C** Survival plot of 3 risk groups classified by Oncotype DX (OS). **D** Hazard ratio of high and intermediate-risk groups using low-risk as baseline (OS). Multivariable Cox models were adjusted for tumor stage, lymph node status, breast cancer subtype (ER+HER2− or ER+HER2+), and patients’ age. *P* values below 0.05 were considered significant

To confirm the prognostic value of the Oncotype DX test, we evaluated BCSS of ER+ patients in the three risk-recurrence groups. As shown, patients in the high-risk group had significantly worse BCSS compared to the intermediate- and the low-risk groups; the intermediate-risk group had significantly worse BCSS than the low-risk group (Fig. 4A, Additional file 1 – Figure 2A). Multivariable Cox regression analysis adjusting for age, stage, grade, lymph node status, and breast cancer subtype also showed consistently worse BCSS for high-risk patients in all years except for 2004 (presumably due to small patient number) and 2015 (presumably due to short follow-up time) with HR ranging from 3.25 to 10.36 compared to low-risk patients (Fig. 4B). For intermediate-risk patients, significantly worse BCSS was observed in the majority of evaluated years but with much lower HRs (Fig. 4B). Overall, the high- and intermediate-risk patients showed approximately sixfold and twofold higher risk of decreased BCSS, respectively, compared to patients in the low-risk group. When combining all years, an identical observation was made; both the intermediate and high-risk patients had significantly shorter BCSS compared to low-risk patients in a multivariable Cox regression model (Additional file 2 – Table 1). Similar findings were obtained using OS instead of BCSS, but the degree of significance tended to be lower (Fig. 4C, D, Additional file 1 – Figure 2B, Additional file 2 – Table 2).

### Chemotherapy improves the prognosis of high-risk patients identified by the Oncotype DX test

Oncotype DX has been used to decide the use of adjuvant chemotherapy in ER+ breast cancer. We thus performed multivariable Cox regression analysis to examine whether chemotherapy improved the prognosis of patients. As shown in Table 2, we found that high-risk patients benefited from chemotherapy with the risk of overall death decreasing by 40% after chemotherapy use as compared to high-risk patients with no/unknown chemotherapy use (HR = 0.60, *p* = 7.8E−5, Table 2). Patients with intermediate-risk also benefited from chemotherapy use compared to intermediate-risk patients with no/unknown chemotherapy use (HR = 0.74, *p* = 2.8E−4, Table 2). In contrast, chemotherapy was not associated with prolonged OS for patients with low-risk (*p* > 0.05, Table 2).

**Table 2:** Chemotherapy increases overall survival in high-risk score patients.

	High		Intermediate		Low	
	HR	***P*** value	HR	***P*** value	HR	***P*** value
**All (2004–2009)**						
Chemotherapy (yes vs no/unknown)	0.60 (0.47, 0.77)	7.40E−05	0.74 (0.62, 0.87)	2.70E−04	0.90 (0.7, 1.15)	0.40
Radiation (yes vs no/unknown)	1.22 (0.95, 1.56)	0.12	0.72 (0.62, 0.83)	1.40E−05	0.76 (0.65, 0.88)	2.00E−04
Age (old vs young)	1.87 (1.37, 2.55)	7.50E−05	2.39 (1.94, 2.93)	9.36E−17	3.42 (2.75, 4.27)	1.05E−27
Stage (Stage I as baseline)						
Stage II	1.77 (1.35, 2.31)	3.00E−05	1.7 (1.43, 2.02)	3.00E−09	1.53 (1.29, 1.82)	1.10E−06
Stage III	3.42 (1.62, 7.25)	1.30E−03	4.59 (2.67, 7.88)	3.40E−08	2.91 (1.39, 6.08)	4.54E−03
Stage IV	6.68 (2.70, 16.53)	4.00E−05	9.43 (4.86, 18.31)	3.30E−11	21.58 (8.84, 52.64)	1.50E−11
Grade (Grade I as baseline)						
Grade II	1.26 (0.67, 2.34)	0.47	1.29 (1.05, 1.58)	0.01	1.34 (1.14, 1.58)	4.10E−04
Grade III	1.38 (0.75, 2.54)	0.30	1.85 (1.47, 2.32)	1.40E−07	1.28 (0.98, 1.66)	0.07
Lymph node (+ vs −)	1.8 (1.23, 2.64)	2.30E−03	1.07 (0.82, 1.39)	0.64	1.08 (0.83, 1.4)	0.56
**ER+HER2**− **(2010–2015)**						
Chemotherapy (yes vs no/unknown)	0.62 (0.47, 0.82)	7.70E−04	0.79 (0.66, 0.95)	0.01	0.73 (0.52, 1.01)	0.06
Radiation (yes vs no/unknown)	0.84 (0.64, 1.09)	0.19	0.7 (0.59, 0.83)	3.30E−05	0.77 (0.66, 0.89)	4.20E−04
Age (old vs young)	1.37 (0.98, 1.93)	0.07	2.69 (2.07, 3.5)	1.70E−13	3.71 (2.85, 4.82)	1.17E−22
Stage (Stage I as baseline)						
Stage II	1.56 (1.15, 2.11)	3.97E−03	1.44 (1.17, 1.77)	6.60E−04	1.48 (1.24, 1.77)	2.10E−05
Stage III	5.60 (3.05, 10.29)	2.60E−08	2.44 (1.45, 4.11)	7.70E−04	2.86 (1.77, 4.61)	1.70E−05
Stage IV	11.59 (5.82, 23.07)	3.10E−12	19.49 (10.76, 35.28)	1.03E−27	14.44 (7.36, 28.33)	8.10E−15
Grade (Grade I as baseline)						
Grade II	1.83 (0.74, 4.53)	0.19	1.13 (0.9, 1.42)	0.29	1.1 (0.94, 1.3)	0.24
Grade III	1.99 (0.81, 4.85)	0.13	1.86 (1.45, 2.39)	1.10E−06	1.31 (1.01, 1.69)	0.05
Lymph node (+ vs −)	1.37 (0.97, 1.93)	0.08	1.41 (1.11, 1.79)	4.88E−03	1.06 (0.85, 1.32)	0.59
**ER+HER2+ (2010–2015)**						
Chemotherapy (yes vs no/unknown)	0.48 (0.19, 1.25)	0.13	0.5 (0.15, 1.66)	0.26	0.61 (0.13, 2.81)	0.53
Radiation (yes vs no/unknown)	0.68 (0.26, 1.76)	0.42	0.33 (0.1, 1.08)	0.07	0.61 (0.21, 1.77)	0.37
Age (old vs young)	3.75 (0.5, 28.38)	0.20	NA	NA	NA	NA
Stage (Stage I as baseline)						
Stage II	1.28 (0.45, 3.59)	0.64	0.33 (0.04, 2.64)	0.30	0.69 (0.15, 3.19)	0.63
Stage III	2.55 (0.23, 28.81)	0.45	0 (0, Inf)	1.00	0 (0, Inf)	1.00
Stage IV	7.08 (0.68, 73.70)	0.10	NA	NA	0 (0, Inf)	1.00
Grade (Grade I as baseline)						
Grade II	NA	NA	3.32 (0.41, 26.98)	0.26	1.04 (0.32, 3.42)	0.95
Grade III	NA	NA	3.62 (0.4, 33.04)	0.25	0.75 (0.08, 6.82)	0.80
Lymph node (+ vs −)	1.03 (0.26, 4.13)	0.96	4.26 (0.38, 47.95)	0.24	1.33 (0.18, 9.81)	0.78

When we restricted our analysis to ER+HER2− breast cancer, the results similarly indicated a protective effect of chemotherapy in the high-risk (HR = 0.62, *p* = 8.0E−4, Table 2) and intermediate-risk group (HR = 0.79, *p* = 0.01, Table 2). Again, no association between chemotherapy and OS was observed in the low-risk group. For patients with ER+HER2+ breast cancer, identical trends were observed (Table 2). Similar findings were also observed using BCSS although no significant relationship was observed between prolonged BCSS and chemotherapy usage in the intermediate-risk group (Additional file 2 – Table 1).

Interestingly, radiation therapy was protective in patients with intermediate or low Oncotype DX scores but not in patients with high Oncotype DX scores (Table 2). In intermediate-risk patients, an HR of 0.72 (*p* = 1.5E−5) suggested that patients who underwent radiation therapy had longer OS compared to patients who did not undergo radiation therapy. Similarly, an HR of 0.76 (*p* = 2.0E−4) in low-risk patients suggested prolonged OS after the use of radiotherapy (Table 2). A similar observation was made when using BCSS although the association in low-risk patients did not reach statistical significance (Additional file 2 – Table 1).

## Discussion

In this study, we investigated the temporal trends of Oncotype DX usage the first decade after the introduction of the Oncotype DX test in the USA. We found that Oncotype DX was mostly used by ER+ breast cancer patients with negative lymph node status, who were between 45 and 60 years of age. Compared to non-users, Oncotype DX users tended to have significantly longer breast cancer-specific and overall survival after adjustment of clinical factors. We observed an inverse trend between Oncotype DX usage and chemotherapy, which was mainly driven by the decreased chemotherapy rate in patients with low recurrence-risk test results. Survival analyses validated that high-risk patients had significantly worse survival than patients in other risk groups. Moreover, our results indicated that only high-risk but not intermediate or low-risk patients benefit from chemotherapy. These results provide useful information to refine the guidelines for directing Oncotype DX tests.

The current NCCN guidelines for eligible patients include ER+, early-stage (T1 or T2), lymph node-negative (pN0) breast cancer patients [14]. We observed that the majority of performed Oncotype DX tests followed these guidelines, but also noted a slight increase in the use of Oncotype DX among later breast cancer stages and lymph node-positive breast cancer. Based on our survival analysis, both lymph node-negative and positive patients had improved survival after undergoing Oncotype DX testing (Fig. 3). A number of studies have indeed suggested the clinical benefit of Oncotype DX test results in treatment decision-making for lymph node-positive breast cancer [32–34]. Indeed, early results from the large RxPONDER (NCT01272037) trial [13] have indicated that ER+HER2− patients with early-stage, lymph node-positive breast cancer with an Oncotype Dx score < 25 can safely forgo adjuvant chemotherapy.

We have shown that Oncotype DX testing is associated with decreased adjuvant chemotherapy usage and increased survival. Even after adjusting for several clinical variables, including tumor stage and lymph node status, this association between Oncotype DX usage and survival was still significant and suggested an 40% lower risk of breast cancer-specific death upon Oncotype DX usage. While the current study did not address changes in treatment recommendations by physicians/patients after obtaining Oncotype DX results, we hypothesize that altered decision-making underlies this survival benefit. It has been reported that treatment recommendations are reconsidered in ~ 30–45% of patients after Oncotype DX test results became available [16, 29, 30, 35–38]. In the majority of these cases, adjuvant chemotherapy was withheld after reconsideration. Consistently, BCSS was significantly shorter in low risk patients treated with chemotherapy (Additional file 2 – Table 1), suggesting a hazardous role for chemotherapy in this subset of patients.

In this cohort, 6.8% of ER+ breast cancers had HER2+ breast cancer. Oncotype DX testing is not recommended for ER+HER2+ breast cancer [14] due to the availability of HER2-specific therapies for this group of patients. However, approximately 4.7% of ER+HER2+ patients underwent Oncotype DX testing. These patients are a special subset of all ER+HER2+ patients who were HER2+ according to SEER standards but were HER2− based on the Oncotype DX assay. This group of patients had lower rates of chemotherapy usage and did not obtain significant benefits from Oncotype DX testing in terms of OS and BCSS. Consequently, the Oncotype DX test might not be as beneficial to this subset of ER+HER2+ breast cancer compared to ER+HER2− breast cancer.

Although our study provides valuable insights into the use of Oncotype DX testing in breast cancer, some limitations should be noted. First, it is likely that additional patients’ characteristics are associated with Oncotype DX usage besides the variables that we evaluated in this study. For example, marital and insurance status have previously been associated with Oncotype DX usage [24]. Second, the usage of chemotherapy and radiation therapy tends to be underreported in the SEER cohort. For example, a number of patients had “no/unknown” chemotherapy and radiation status, which means that a subset of these patients likely did receive either of these therapies but was classified as “unknown”. This might have affected some of our results. Lastly, our results pertaining to ER+HER2+ breast cancer patients need to be interpreted with caution since this group of patients is likely to represent a subset of ER+HER2+ patients.

## Conclusion

We have provided a comprehensive temporal overview of the use of Oncotype DX in breast cancer patients in the first decade after Oncotype DX was introduced. Our results suggest that the use of Oncotype DX is steadily increasing in ER+HER2− breast cancer. The provided risk scores provide valuable information for patient prognosis and help guide treatment decisions.

## Supplementary Information

**Additional file 1: Supplementary Figures.** Two supplementary figures.

**Additional file 2: Supplementary Tables.** Two supplementary tables.

## Data Availability

All data utilized in this study are publicly available.

## Abbreviations

ASCO: American Society of Clinical Oncology
BCSS: Breast cancer-specific survival
ER: Estrogen receptor
HR: Hazard ratio
HER2: Human epidermal growth factor receptor 2
NCCN: National Comprehensive Cancer Network
OS: Overall survival
pN+: Lymph node-positive
pN0: Lymph node-negative
PR: Progesterone receptor
SEER: Surveillance, Epidemiology, and End Results
