# Supplementary material for: Impact of Oncotype DX testing on ER+ breast cancer treatment and survival in the first decade of use
Source: Breast Cancer Res. 2021 Jul 17;23:74. doi: 10.1186/s13058-021-01453-4 (PMC8285794; doi:10.1186/s13058-021-01453-4)
Supplement: Supplementary file 1 — Additional file 1: Supplementary Figures. Two supplementary figures. [file 13058_2021_1453_MOESM1_ESM.pdf]

Supplementary Figures

Impact of Oncotype DX testing on ER-positive breast cancer treatment and survival in the first decade of use

Evelien Schaafsma, Baoyi Zhang, Merit Schaafsma, Chun-Yip Tong, Lanjing Zhang, Chao Cheng

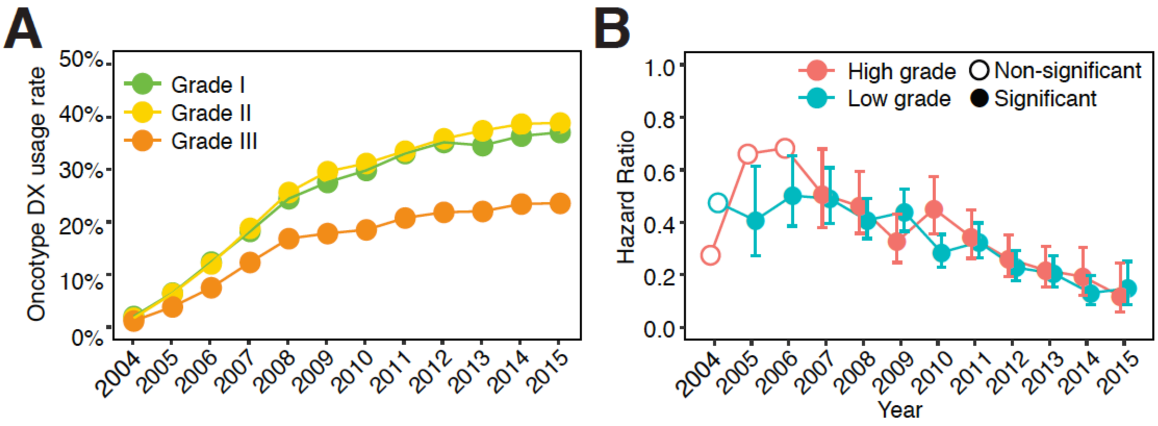

Suppl. Figure 1. **Oncotype DX usage rates and hazard ratios stratified by grade. A.** Oncotype DX usage rate among the three breast cancer grades. Usage rates were calculated based on the total number of patients within each indicated grade group. **B.** Hazard ratios of Oncotype DX usage stratified by tumor grade between 2004 and 2015 from univariate Cox regression models.

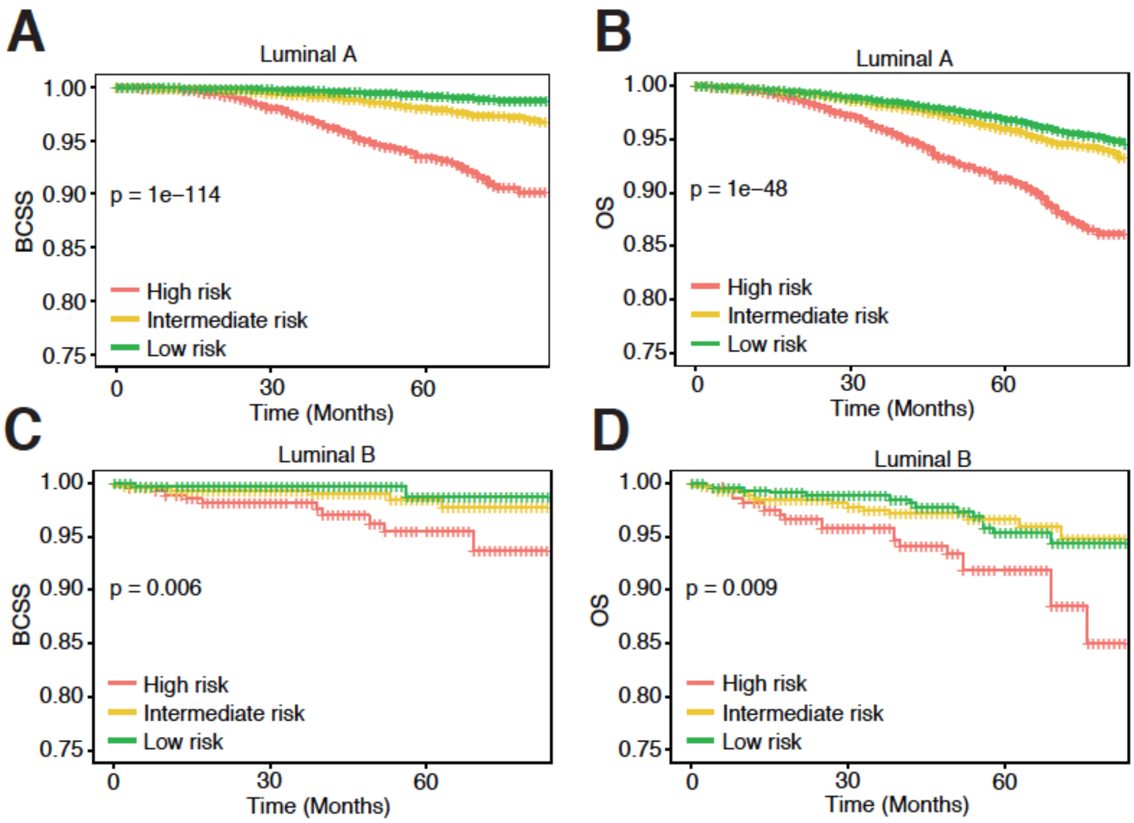

Suppl. Figure 2. **Oncotype DX score is predictive of patient's survival in Luminal A and Luminal B breast cancer. A.** Survival plot of 3 risk groups classified by Oncotype DX (BCSS) in Luminal A breast cancer. **B.** Survival plot of 3 risk groups classified by Oncotype DX (OS) in Luminal A breast cancer. **C.** Survival plot of 3 risk groups classified by Oncotype DX (BCSS) in Luminal B breast cancer. **D.** Survival plot of 3 risk groups classified by Oncotype DX (OS) in Luminal B breast cancer.
